# Mental Health Impact of COVID Pandemic on Veterans Transitioning from Military

**DOI:** 10.1007/s11414-023-09869-w

**Published:** 2023-12-22

**Authors:** Gary R. Bond, Monirah Al-Abdulmunem, Daniel R. Ressler, Robert E. Drake

**Affiliations:** 1 Social Policy and Economics Research, Westat, Inc., New Hampshire Office, Wheeler Professional Park, 1 Oak Ridge Road, Building 3, Second Floor, Suite 2 West, West Lebanon, NH 03784 USA; 2 Department of Psychiatry, Columbia University College of Physicians and Surgeons, New York, NY USA

## Introduction

The COVID pandemic led to a sharp increase in anxiety, depression, stress, substance use, and other behavioral health conditions in the USA.^1–3^ The increase was greatest during the early months of the COVID pandemic, starting in March of 2020. US Census Bureau data collected in April and May 2020 indicated a threefold increase of positive screens for depressive and/or anxiety disorders in the general US adult population compared to 2019.^4^ Other studies also detected sharp increases in depressive symptoms during early stages of the pandemic compared to pre-pandemic. For example, one longitudinal study of a nationally representative group of US adults using a widely used depression scale, the Patient Health Questionnaire-9 (PHQ-9),^5^ found that the percentage of respondents screened with at least moderate depression increased from 5.7% pre-pandemic to 14.8% during the pandemic.^6^

The COVID pandemic severely impacted the US work force, leading to lay-offs and changes where Americans worked. During the spring and summer of 2020, the US labor market experienced a major shock, and the monthly unemployment rate did not return to pre-pandemic levels until late 2021.^7^ Surveys found that workers who lost their jobs during the COVID-19 pandemic developed behavioral health conditions.^8, 9^

Researchers have also examined the mental health impact of the COVID pandemic on US veterans. A 3-year longitudinal study examined self-reported psychiatric symptoms (i.e., positive screens for major depressive disorder, generalized anxiety disorder, or posttraumatic stress disorder) in a national representative sample of 2289 veterans.^10^ Participants completed interviews before, during, and 2 years after the height of the pandemic (Fall 2019, Fall 2020, and Summer 2022, respectively). Similar to surveys in the general population, the veteran sample reported increased psychiatric symptoms during the pandemic: Positive screens for any psychological distress increased from 8.5% before the pandemic to 12.8% during the pandemic and decreased to 7.5% in the period after the height of the pandemic. However, 84% of veterans were *resistant*; that is, they screened negative for psychiatric symptoms at all time periods.

Another veteran study examined changes in psychiatric symptoms from pre-pandemic to during the pandemic.^11^ Using a nationally representative, prospective cohort survey of 3078 veterans, the authors found an increased prevalence in generalized anxiety disorder, but no significant changes in prevalence of major depressive disorder or posttraumatic stress disorder. Overall, the sample reported a 13% increase in psychological distress, which was considered a clinically meaningful increase.

A further study examined the longitudinal impact of the pandemic on 398 veterans receiving outpatient mental health care.^12^ Comparing changes from pre-pandemic to during the pandemic in the summer of 2020, pandemic-related stressors, consisting of negative life changes in eight life domains (e.g., work, home life, and social activities), were associated with increased mental and physical health distress. Positive behavioral adaptations (such as increased social connections with family and friends, physical exercise, helping other people, and finding greater purpose and productivity during life pursuits) lessened the negative impact of the stressors.

The current report examined the impact of the COVID pandemic on a group of veterans undergoing the transition from military to civilian life. The study sample consisted of young, enlisted men and women with service-connected disabilities who volunteered to participate in a study evaluating a new program to help transitioning veterans gain meaningful employment. While many service men and women successfully make the transition from the military, a large literature has documented that a subgroup experience stress related to the challenges of finding employment, changes in daily schedules, home life, and income, as well as all aspects of the shift from military to civilian culture.^13–17^ As many as two-thirds of veterans report significant difficulty transitioning from the military.^18^ Transition stresses can lead to elevated levels of psychiatric symptoms.^19–22^ For many veterans, securing a job that matches their skills and interests is the most significant source of stress during the transition period.^23–26^ In addition, the first civilian job is not always a good fit. Job turnover is very high among veterans during the first two years after leaving the military.^27^ Veterans who terminate from civilian jobs during the transition period experience increased risk for negative mental health outcomes.^28^ The impact of the COVID pandemic on transitioning veterans has not been reported in the literature.

As part of the evaluation study assessing an innovative employment program for transitioning veterans, study participants were interviewed before and during the COVID pandemic.^29^ The survey included standardized measures of self-reported psychiatric symptoms, including depression. Overall, 41.9% of the sample of 229 veterans completing baseline interviews screened positive for moderate depression, a rate that was four times greater than in a national representative sample of veterans (10.3%).^30^ Because study participants completed follow-up interviews after enrollment, the serendipitous timing of the interview schedule offered an opportunity to assess the impact of the COVID pandemic by comparing changes over time. Moreover, the sample included a subgroup who indicated that the pandemic had affected their mental health. The current report examined the longitudinal impact of the pandemic on two groups — veterans reporting that the pandemic influenced their mental health and those who reported that they were relatively unaffected.

In a secondary analysis of the longitudinal data from the evaluation study, the authors proposed three exploratory hypotheses based on published studies in both civilian and veteran samples. The authors hypothesized that (1) the total veteran sample would report increased depression during the pandemic compared to pre-pandemic levels and that (2) this increase would be greater for veterans reporting that the pandemic had affected their mental health than those who did not. A final hypothesis examined the protective effects of employment, namely, that (3) veterans gaining employment during the pandemic would experience less adverse impact on their mental health. This hypothesis was derived from a large literature supporting a positive correlation between employment and mental health.^31, 32^

## Methods

### Overview

This report is a secondary analysis of data from a randomized controlled trial evaluating an innovative employment program for transitioning veterans with service-connected disabilities seeking employment.^29^ Using the PHQ-9, the study examined changes from baseline to two years in the rate of positive screens for moderate depression. The analyses compared veterans indicating that the pandemic had affected their mental health to those who did not. In addition, because of the common goal of employment for veterans enrolling in the study, the impact of employment status during the height of the pandemic was also examined. The Westat Institutional Review Board approved the study, which followed the principles outlined in the Declaration of Helsinki.

### Study sample and data collection

The parent study enrolled participants meeting the following criteria: enlisted men and women transitioning from the military and seeking employment, under the age of 45, at least 6 months of active military service with an Honorable or General discharge, within 6 months before separation or 12 months after separation, either without civilian employment (before separation) or unemployed or working in temporary jobs (after separation), and receiving or applying for a service-connected disability rating and compensation through the Veterans Benefits Administration.

Trained interviewers conducted all research interviews by telephone. Enrollment procedures included email contact initiated by prospective participants, a screening interview, informed consent, and study enrollment, followed by the baseline interview. Follow-up interviews with a battery of outcome measures were conducted at 1 and 2 years. Employment outcomes were assessed at these interviews and in brief interviews at 4 and 8 months between annual interviews.

Participants completed their baseline assessments before the onset of the COVID-pandemic (between May 2018 and June 2019) and then completed 2-year follow-up interviews during the height of the pandemic or shortly post onset. In interviews conducted between August 2020 and December 2021, participants also responded to a new checklist, the COVID Pandemic Checklist, assessing how the pandemic impacted them. The study sample for the current secondary analysis consisted of participants completing the checklist.

### Measures

#### Participant characteristics

The baseline interview included demographic characteristics, military history, and background information.

#### Patient Health Questionnaire-9 (PHQ-9)

The PHQ-9 is a 9-item self-report depression checklist that has been well validated in two large studies and has been used in many medical surveys.^5^ It is also used in routine clinical practice as a screener for depression. According to the scale’s developers, a score of 10 or more indicates moderate depression. The internal consistency coefficient (Cronbach’s alpha) for PHQ-9 was 0.88 in the study sample.

#### COVID pandemic checklist

Because of the pandemic, the research team added a new checklist consisting of 14 questions about participants’ experience with the COVID pandemic. Three questions referred to the impact of pandemic on employment (employment status, work schedule, and wages), eight items concerned the impact of the pandemic on other life domains (e.g., mental health, changes in substance use, COVID infection), three items concerned future plans, and one item was a global rating of concern about the pandemic.

#### Employment rates

The current study examined employment status (currently employed/not employed) during the same interview when the COVID Pandemic Checklist was completed.

### Statistical analyses

In addition to familiar descriptive statistics (means, standard deviations, frequencies, and percentages), the analyses included conventional univariate tests of significance (chi squares and *t* tests).

## Results

### Sample characteristics

The study sample for the COVID analysis consisted of 109 participants who completed the COVID Pandemic Checklist as well as the baseline and two-year follow-up interviews. A checklist item asking participants whether the pandemic affected their mental health was used to define two subgroups: the affected mental health group consisting of 41 veterans who reported COVID affected their mental health and the unaffected mental health group consisting of 68 who reported being unaffected by COVID in terms of their mental health. Table 1 includes sample characteristics of the total sample in addition to these two groups.

**Table 1:** Baseline characteristics for transitioning veterans with service-connected disabilities

	Affected mental health group (*N* = 41)	Unaffected mental health group (*N* = 68)	Total sample (*N* = 109)	Significance (affected vs. unaffected)
Age, *M (SD)*	31.6 (6.2)	31.7 (6.8)	31.7 (6.5)	*t* = .14, *p* = .89
Gender, *n (%)*				
Male	31 (75.6%)	58 (85.3%)	89 (81.7%)	*χ*^2^ = 1.06, *p* = .30 (male vs. female)
Female	9 (22.0%)	10 (14.7%)	19 (17.4%)	
Non-binary	1 (2.4%)	0 (0.0%)	1 (0.9%)	
Marital status, *n (%)*				
Married	22 (53.7%)	49 (72.1%)	71 (65.1%)	*χ*^2^ = 3.81, *p* = .05
Unmarried	19 (46.3%)	19 (27.9%)	38 (34.9%)	
Race, *n (%)*				
White	16 (39.0%)	31 (45.6%)	47 (43.1%)	*χ*^2^ = .45, *p* = .50 (White vs. non-White)
Black	16 (39.0%)	21 (30.9%)	37 (33.9%)	
Asian	1 (2.4%)	7 (10.3%)	8 (7.3%)	
Other	8 (19.5%)	9 (13.2%)	17 (15.6%)	
Hispanic, *n (%)*	9 (22.0%)	8 (11.8%)	17 (15.6%)	*χ*^2^ = 2.02, *p* = .16
Education, *n (%)*				
High school diploma/GED	3 (7.3%)	4 (5.9%)	7 (6.4%)	*χ*^2^ = .13, *p* = .72 (associate’s or lower vs. bachelor’s or higher)
Technical certificate, some college, or associate’s degree	24 (58.5%)	43 (63.2%)	67 (61.5%)	
Bachelor’s degree or higher	14 (34.1%)	21 (30.9%)	35 (32.1%)	
Active duty status at baseline, *n (%)*				
Veteran	34 (82.9%)	42 (61.8%)	76 (69.7%)	*χ*^2^ = 5.43, *p* = .02
Active military	7 (17.1%)	26 (38.2%)	33 (30.3%)	
Military branch, *n (%)*				
Army	29 (70.7%)	46 (67.6%)	75 (68.8%)	*χ*^2^ = .11, *p* = .74 (Army vs. other)
Air Force	6 (14.6%)	9 (13.2%)	15 (13.8%)	
Navy	5 (12.2%)	9 (13.2%)	14 (12.8%)	
Marine Corps	1 (2.4%)	3 (4.4%)	4 (3.7%)	
Coast Guard	0 (0.0%)	1 (1.5%)	1 (0.9%)	
Served in a combat zone, *n (%)*	24 (58.5%)	34 (50.0%)	58 (53.2%)	*χ*^2^ = .75, *p* = .39
Disability rating, *M (SD)*	77.2% (22.5) (*N* = 39)	71.6% (23.3) (*N* = 67)	73.7% (23.0) (*N* = 106)	*t* = 1.21, *p* = .23

Participants had a mean age of 31.7; 82% were male, 43% White, 34% Black, and 16% Hispanic; and 65% were married. In terms of education at baseline, 62% had completed an associate’s degree, a technical certificate, and/or some college. At baseline, 70% had terminated from the military while the remainder were active-duty personnel who were about to leave the military. Over half of the sample were enlisted in the Army and a little over half served in a combat zone. The mean service-connected disability rating reported by participants was 73.7%. (Disability ratings ranged from 0 to 100%.) When comparing the characteristics of those who reported that their mental health was affected by COVID and those who were not, the samples did not differ on any characteristics, except marital status (unmarried veterans were more likely to be affected) and active-duty status at baseline (veterans who were on active duty at baseline were less likely to be affected). Over the 2-year follow-up period, 102 (94%) worked in a paid job, and 68 (62%) were employed at the time of the 2-year interview.

To test for non-response bias, the study sample, comprised of the COVID checklist respondents (*N* = 109), was compared to non-respondents (*N* = 120) on baseline characteristics. (Results not shown.) Respondents were older, more often married, and better educated, and they reported significantly greater life satisfaction, less depression, and less financial distress than non-respondents.

### Impact of COVID

Of 109 participants, 29 (27%) reported a negative impact on their employment status (job search more difficult, furloughed, laid off, job start delayed or discontinued because of pandemic), as shown in Table 2. Most employed participants reported no changes to their work schedule or wages due to the pandemic. Among 64 employed veterans, 40 (63%) worked at their office/job site, while 24 (38%) worked remotely, mostly due to the pandemic.

**Table 2:** Participants’ experience during COVID pandemic (August 2020 to December 2021)

Effect of COVID-19 pandemic on employment situation (*N* = 109)	
Continuing to work at job site	40 (36.7%)
Already working remotely, no change	4 (3.7%)
Working remotely since the pandemic, little change	20 (18.3%)
Furloughed	5 (4.6%)
Laid off	5 (4.6%)
Job start delayed	4 (3.7%)
Job search has been more difficult	13 (11.9%)
Discontinued job search because of the pandemic	2 (1.8%)
Wasn't interested in or looking for work	6 (5.5%)
Other	6 (5.5%)
No response	4 (3.7%)
Change in work schedule	
Yes, hours increased	4 (3.7%)
Yes, hours decreased	8 (7.3%)
Yes, schedule changed, but same number of hours	2 (1.8%)
No change due to pandemic	67 (61.5%)
Not working	28 (25.7%)
Change in wages	
Yes, wage increased	3 (2.8%)
Yes, wage decreased	6 (5.5%)
No change due to pandemic	72 (66.1%)
Not working	28 (25.7%)
Effect of pandemic on other aspects of living (*N* = 109)	
Physical health has been affected	12 (11.0%)
Mental health has been affected	41 (37.6%)
Drinking alcohol or using recreational drugs more often	5 (4.6%)
Drinking alcohol or using recreational drugs less often	0 (0.0%)
I have been infected by the virus	8 (7.3%)
Family member has been infected by the virus	22 (20.2%)
I know someone else personally who has been infected by the virus	20 (18.3%)
Social isolation	6 (5.5%)
Other	11 (10.1%)
No response/no effect	52 (47.7%)
Effect of pandemic on future plans (*N* = 109)	
Planning to go back to school	11 (10.1%)
Considering new career path	11 (10.1%)
Other^a^	57 (52.3%)
No change in future plans	48 (44.0%)
No response	4 (3.7%)
Overall rating of concern about the pandemic (*N* = 109)	
Very concerned	23 (21.1%)
Somewhat concerned	55 (50.5%)
Somewhat unconcerned	19 (17.4%)
Very unconcerned	12 (11.0%)

^a^43 (75.4%) identified “travel, social gatherings,” 5 (8.8%) identified “delay in job start,” 3 (5.3%) identified “difficulty meeting education goals,” and 2 (3.5%) identified “delay in obtaining a house.” No other change mentioned by more than one respondent

On the item rating on overall concern about the COVID pandemic, 78 (72%) of participants indicated that they were “very concerned” or “somewhat concerned.” However, when asked to identify which of eight life domains that potentially might be affected (e.g., physical health, increase alcohol, social isolation) that might be affected, in all but one life domain, participants indicated that they experienced relatively little impact. The one exception was mental health; 41 (38%) reported that the pandemic affected their mental health.

### Rates of depression before and during COVID

Table 3 shows the rate of moderate depression (PHQ-9 score 10 or higher) at baseline and 2-year follow-up. Contrary to the first hypothesis, the total sample had a nonsignificantly higher rate who tested positive for moderate depression at baseline (33.0%) than at 2-year follow-up (27.5%). Similarly, the rate of depression in the unaffected mental health group was higher at baseline (23.5%) than at 2 years (14.7%), while the affected mental health group had the same frequency at both time points (49.0%). Neither the total sample nor the two subgroups had a significant change in the rate of depression between these time points.

**Table 3:** Changes in rate of moderate depression among transitioning veterans from pre-pandemic to pandemic period

	Screened positive for moderate depression			PHQ-9 total scores		
	Baseline (pre-pandemic)	Two years (during pandemic)	Significance (McNemar’s test)	Baseline (pre-pandemic)	Two years (during Pandemic)	Significance
	*N* (%)	*N* (%)		*M* (SD)	*M* (SD)	
Total sample (*N* = 109)	36 (33.0%)	30 (27.5%)	*p* = .33	7.35 (5.81)	7.41 (5.91)	*t* = .14, ns
Pandemic affected mental health (*N* = 41)	20 (48.8%)	20 (48.8%)	*p* = 1.00	9.88 (6.56)	10.37 (6.19)	*t* = .58, ns
Pandemic did not affect mental health (*N* = 68)	16 (23.5%)	10 (14.7%)	*p* = .21	5.88 (4.74)	5.63 (4.98)	*t* = − .34, ns
Significance	*χ*^2^ = 7.37, *p* < .01	*χ*^2^ = 14.89, p < .001		*t* = − 3.45, *p* < .01	*t* = − 4.15, *p* < .001	

Changes in depression were also examined using PHQ-9 scores, as shown in Table 3. In the total sample, the mean PHQ-9 score did not significantly increase between baseline and 2 years. The same held true in the affected mental health group and the unaffected mental health group. However, the affected group had significantly higher depression scores than the unaffected group both at baseline and 2 years.

### Employment rates for affected and unaffected groups

At the time of the COVID Pandemic Checklist was completed, 22 (54%) in the affected group were employed compared to 47 (69%) in the unaffected group, *χ*^2^ = 1.06, *p* = 0.30. Thus, the hypothesis that employment would to be a protective factor mitigating the impact of the COVID pandemic was not confirmed.

## Discussion

In a secondary analysis of interviews from a study evaluating the effectiveness of an innovative employment program, over 70% of a group of transitioning veterans with service-connected disabilities indicated personal concerns about their day-to-day living due to the COVID pandemic. Participants indicated that among all life domains, mental health was the most impacted. One-third reported that the COVID pandemic had affected their mental health.

During the pre-pandemic period, the rate of moderate depression found in this sample of transitioning veterans was six times the rate found in a national representative sample of the general population (33.0% versus 5.6%).^6^ However, the percentage of transitioning veterans screening positive for moderate depression did not increase during the pandemic, unlike that national representative sample, which increased more than two-fold. Nevertheless, the rate of moderate depression during the pandemic was three times higher in the subgroup whose mental health was affected by the pandemic than in the national civilian sample. The comparisons were quite different for the group indicating that their mental health was unaffected by the pandemic. During the pandemic, the rate of moderate depression was very similar in the unaffected veteran sample compared to the civilian sample.

The hypothesis that the pandemic would lead to increased levels of depression in transitioning veterans with service-connected disabilities was not confirmed. Nor was the hypothesis upheld in the subgroup indicating that the pandemic had affected their mental health. The apparent lack of impact of the pandemic on depression levels in the current sample of transitioning veterans, even among those who indicated that the pandemic had affected their mental health, was surprising, given the statistically significant impact of the pandemic on a nationally representative veteran sample. These findings also contradict researchers’ prediction in the early days of the pandemic that it would create a “perfect storm” for veterans with pre-existing mental health conditions, resulting in exacerbation of psychiatric symptoms.^33^

While many factors may have affected the current findings, our interpretation is that substantive differences (that is, characteristics of the sample), rather than methodological differences (such as the timing of the surveys or measures used), best explain the findings. As discussed below, possible explanations include pre-existing depressive symptoms, military service (including the contemporaneous experience of transitioning to civilian life and high levels of service-connected disability), young age, and employment.

### Pre-existing depressive symptoms

The parsimonious explanation for the apparent lack of impact of the pandemic on depressive symptoms is that depressive symptoms were already prevalent in the study sample of transitioning veterans prior to the pandemic. The relative absence of a COVID impact on mental health may have been a result of a ceiling effect in which those who were vulnerable to depression were already experiencing depression related to the stresses of transitioning, therefore masking any incremental impact of the pandemic.

### Military service

Most transitioning veterans — particularly those who indicated that their mental health was not affected by the pandemic — may have been resilient or hardened due to the stress of their military experience and service-connected disabilities. Military training and experience emphasizes overcoming adversity and maintaining the mission in the face of personal hardship. Some veterans developed coping strategies making them more resilient to pandemic-related stressors.^12^

### Young age

The current study sample of transitioning veterans differed substantially on many demographic measures from samples used in earlier veteran studies of the impact of the COVID pandemic.^10, 11^ For example, the mean age was over 60 years in national representative veteran samples in these two previous veteran studies examining the mental health impact of COVID, whereas the mean age of participants in the current study was 32 years. The COVID pandemic may have had a more severe mental health impact on older people because of their greater vulnerability to the coronavirus and especially to the risk for hospitalization and death.

The protective and risk factors of age for psychological impact of COVID are likely complex, however. The two previous veteran studies reported seemingly conflicting results for the influence of age on increase in psychological distress during the pandemic. One study found that veterans aged 18 to 44 years (similar to the age range for the current study sample) evidenced the largest increases in distress from pre-pandemic to during the pandemic,^10^ whereas the other study reported that the subgroup of veterans aged 45–65 accounted for the increased in distress.^11^ Another veteran study found that the increase in suicide ideation during the pandemic was associated with older age.^34^

### Employment

The hypothesis that employment would counteract the negative mental health impact of the pandemic was not supported. An alternative hypothesis is that the pandemic disrupted the mental health benefits of working. During 2020, many workers experienced pandemic-related work-related challenges and stresses,^35^ especially in occupations with increased risk for contracting COVID, such as in the service industry and caretaking professions.^36^

### Study limitations

First, the study sample was small, limiting statistical power. Second, the sample was a volunteer sample who had enrolled in a study that offered help finding employment. Third, selection biases were found at baseline for the study sample compared to non-respondents, further limiting the generalizability of the findings. The study sample differed from the non-respondent sample on several baseline characteristics; notably, they were less depressed than the non-respondent sample. The impact of the pandemic on the mental health of the non-respondent sample is unknown. Fourth, the study used a standard, self-report screen for depression. The PHQ-9 is intended to be followed up by an experienced assessor. A standardized clinical interview conducted by an experienced assessor would have been more rigorous. Fifth, a new questionnaire was used to measure the impact of the pandemic. Because of the time-sensitive nature of the study, it was not feasible to validate the checklist before using it. Finally, possibly the most important study limitation was that the interview schedule was based on the follow-up periods defined by the randomized controlled trial, not the time course for the pandemic. The impact of COVID on participants’ employment and associated mental health symptoms may have been stronger if all these assessments had been conducted during the period from March to mid-summer 2020, when the labor market and the society experienced the greatest shock.^37^

## Implications for Behavioral Health

Young veterans with service-connected disabilities who have recently transitioned from the military often report depression, anxiety, and other mental health symptoms. Surprisingly, the COVID pandemic may have had little or no incremental impact on their depressive symptoms. The alarming rates of behavioral health problems among transitioning veterans have been well documented. They include heightened rates of suicide and opioid use. The parent study from which the current sample was drawn focused on helping veterans finding meaningful work. That focus may have promoted greater resiliency to cope with the pandemic.

## Data Availability

De-identified data from this study and the analytic code used to conduct the analyses are available on the open science platform (https://osf.io/6cdq8/). The current study was secondary analysis of Independence Project, described at https://osf.io/fud8b/.
